# Supplementary material for: Gene-specific mechanisms direct glucocorticoid-receptor-driven repression of inflammatory response genes in macrophages
Source: eLife. 2018 Feb 9;7:e34864. doi: 10.7554/eLife.34864 (PMC5821458; doi:10.7554/eLife.34864)
Supplement: Supplementary file 4. [file elife-34864-supp4.doc]

**Supplementary File 4: primer pairs used in the study**

| mIL1a_F | GGAAGTGCTGACAGTCTGTATGTAC |
| --- | --- |
| mIL1a_R | GTGGCTCCACTAGGGTTTGCTC |
| mIL1a_intron_F | GCAGCAAGGAAAAGGCACATCAGG |
| mIL1a_intron_R | ACGAAGCACTCCACTCCCACAGT |
| mIL1b_F | GGGCTGCTTCCAAACCTTTGACC |
| mIL1b_R | GTAGCTGCCACAGCTTCTCCACAGCC |
| mIL1b_intron_F | AAGGAGACAGCACACACTGCTGC |
| mIL1b_intron_R | AACAAGCATTCCAAGCGCGGTCA |
| mTnf_F | CCAGGATCATCTTCTCAAAATTCGAGTG |
| mTnf_R | TCTAAGAGCTCTGTCTTTTCTCAGCC |
| mCcl2_F | AGGTCCCTGTCATGCTTCTGGG |
| mCcl2_R | CCTCATTGGGATCATCTTGCTGGTG |
| mMyc_F | TTTGAAGGCTGGATTTCCTTTG |
| mMyc_R | ATGGAAGAAAGACTGTCCTAACC |
| mErrfi1_F | TGGATTTCTCCATAATGGTCAGG |
| mErrfi1_R | TATCACTGCAGTATGAGGAACAC |
| mLif_F | ATTGTGCCCTTACTGCTGCTG |
| mLif_R | GCCAGTTGATTCTTGATCTGGT |
| mb-actin_F | AGGTGTGCACTTTTATTGGTCTCAA |
| mb-actin_R | TGTATGAAGGCTTTGGTCTCCCT |
| hp300_F | CAACTTACTGACTGAGCCTCTT |
| hp300_R | CCTCCATCCATCATGACAATACT |
| mTnf TSS_F | GCTATAAAGGCAGCCGTCTGCACAG |
| mTnf TSS_R | GGGAGCTATTTCCAGGATGTTCTGG |
| mTnf -200bp_F | ATTGGCCCCAGATTGCCACAGAATC |
| mTnf -200bp_R | CACCTCTGTCTCGGTTTCTTCTCCAT |
| mIl1b TSS_F | CAGTTTTGTTGTGAAATCAGTTAACCC |
| mIl1b TSS_R | CCTATTAGGCCTCGAACCACTG |
| mIl1b -10kb_F | GGGGCAACACTGGGACTTTCCAAAT |
| mIl1b -10kb_R | AACCAAGGCTGCCAGTCACAGGT |
| mIl1a TSS_F | TGGCCACTCCTACCTGCTTGAGG |
| mIl1a TSS_R | CATAGCCTTAGACGTGATAAGGACAATACC |
| mIl1a -10kb_F | GCGACCTCGAGTCAGTCCTCACT |
| mIl1a -10kb_R | AGCACCAGAAGTGACTCATCCTCCA |
| mIl1a -20kb_F | ACATTCCACTTCCAGCCTTAAA |
| mIl1a -20kb_R | TACTGAGTAATCGCTCTGGATTC |
| mCcl2 TSS_F | GGGTGATGCTACTCCTTGGCACC |
| mCcl2 TSS_R | TCCCGTCTGGCTCTCTGCACTTC |
| mCcl2 -12.5kb_F | CGAACCGGAGCCGTGGTGTT |
| mCcl2 -12.5kb_R | AGGTCCACAGCAGGATGTACCCAC |
| mErrfi1 TSS_F | CCAGATAATGAGGCCAGCACCTC |
| mErrfi1 TSS_R | AGACCTATGGCTGACCTCTGTGG |
| mLif TSS_F | AGGCCACCAACTTCAGACG |
| mLif TSS_R | CTTCAGGGTGACACCATCGTT |
| mCxcl10 -100bp_F | TTTGGAGATGACTCAGCAAGG |
| mCxcl10 -100bp_R | CCAAGTTCATGGGTCACAATAAA |
| mCd40 -450bp_F | TTCTCAAGAAAGAGAAACTGGGT |
| mCd40 -450bp_R | TGTCACCGCAAAGTCAGAAA |
| mTnfsf9 -800bp_F | ACAGTGAAAGAATGGGAAATTGG |
| mTnfsf9 -800bp_R | GTCTCTTAGGACTCCAGAAACAG |
| mTrim13 -1kb_F | ACTCGGCATTTGATTATCGGT |
| mTrim13 -1kb_R | GCTCAATCAGTGTTCGTGTG |
