# Supplementary material for: Gene-specific mechanisms direct glucocorticoid-receptor-driven repression of inflammatory response genes in macrophages
Source: eLife. 2018 Feb 9;7:e34864. doi: 10.7554/eLife.34864 (PMC5821458; doi:10.7554/eLife.34864)
Supplement: Transparent reporting form [file elife-34864-transrepform.docx]

***eLife’s* transparent reporting form**

We encourage authors to provide detailed information *within their submission* to facilitate the interpretation and replication of experiments. If you have any questions, please contact us: [editorial@elifesciences.org](mailto:editorial@elifesciences.org).

**Sample-size estimation**

- You should state whether an appropriate sample size was computed when the study was being designed
- You should state the statistical method of sample size computation and any required assumptions
- If no explicit power analysis was used, you should describe how you decided what sample (replicate) size (number) to use

Please outline where this information can be found within the submission (e.g., page numbers or figure legends), or explain why this information doesn’t apply to your submission:

No explicit power analysis was used prior to experiments. Each experiment was repeated the number of times indicated in Figure Legends.

Sample sizes can be found in Figure Legends and statistical analysis used can be found in Materials and methods.

**Replicates**

- You should report how often each experiment was performed
- You should include a definition of biological versus technical replication
- The data obtained should be provided and sufficient information should be provided to indicate the number of independent biological and/or technical replicates
- If you encountered any outliers, you should describe how these were handled
- Criteria for exclusion/inclusion of data should be clearly stated
- High-throughput sequence data should be uploaded before submission, with a private link for reviewers provided (these are available from both GEO and ArrayExpress)

Please outline where this information can be found within the submission (e.g., page numbers or figure legends), or explain why this information doesn’t apply to your submission:

All ChIP-qPCR and RNA analysis was performed at least three times (the exact number is indicated in Figure Legends for Figs 3-5).

In each case ‘n’ refers to biological replicates. Biological replicates are defined as replicates where different samples, from different mice, were used to measure the same variables.

The significance of difference in pairwise comparisons was evaluated using two-tailed *t* test (see relevant Figure Legends, Figs 3-5) with significance cut-off set at 0.05 or 0.01.

Bioinformatic analysis of NGS data are summarized in the Materials and methods, Results and Figure Legends.

All ChIP-seq metrics are supplied as Supplementary File 2.

All transient transfections experiments and inhibitor studies were performed ≥3 times as stated in Fig. 4B and Fig. 5C-D Legends.

**Statistical reporting**

- Statistical analysis methods should be described and justified
- Raw data should be presented in figures whenever informative to do so (typically when N per group is less than 10)
- For each experiment, you should identify the statistical tests used, exact values of N, definitions of center, methods of multiple test correction, and dispersion and precision measures (e.g., mean, median, SD, SEM, confidence intervals; and, for the major substantive results, a measure of effect size (e.g., Pearson's r, Cohen's d)
- Report exact p-values wherever possible alongside the summary statistics and 95% confidence intervals. These should be reported for all key questions and not only when the p-value is less than 0.05.

All statistical methods can be found in the “*Quantification and Statistical Analysis”* subsection of Materials and methods. All sample sizes are listed in Figure Legends 3-5. P-values and E-values are reported in the figures or corresponding Figure Legends.

Two-tailed *t* test was used for a pairwise comparison of means (Figs 3-5).

Please outline where this information can be found within the submission (e.g., page numbers or figure legends), or explain why this information doesn’t apply to your submission:

(For large datasets, or papers with a very large number of statistical tests, you may upload a single table file with tests, Ns, etc., with reference to page numbers in the manuscript.)

**Additional data files (“source data”)**

- We encourage you to upload relevant additional data files, such as numerical data that are represented as a graph in a figure, or as a summary table
- Where provided, these should be in the most useful format, and they can be uploaded as “Source data” files linked to a main figure or table
- Include model definition files including the full list of parameters used
- Include code used for data analysis (e.g., R, MatLab)
- Avoid stating that data files are “available upon request”

Please indicate the figures or tables for which source data files have been provided:

All raw data used in the making of the graphs for Figs 3-5 are provided as ‘Source data’ Excel files.
